# Galectin-3 and cancer immunotherapy: a glycobiological rationale to overcome tumor immune escape

**DOI:** 10.1186/s13046-024-02968-2

**Published:** 2024-02-06

**Authors:** Giorgia Scafetta, Calogero D’Alessandria, Armando Bartolazzi

**Affiliations:** 1 Pathology Research Laboratory St, Andrea University Hospital, Via Di Grottarossa 1035, 00189 Rome, Italy; 2 Klinikum Rechts Der Isar, Nuclear Medicine Department, Technical University Munich, Ismaninger Str. 22, 81675 Munich, Germany; 3 Pathology Research Laboratory Cancer Center Karolinska, Karolinska Hospital, S-17176 Stockholm, Sweden

**Keywords:** Immunotherapy, Checkpoint inhibitors, Galectin-3, Tumor microenvironment, Predictive markers of immune-response

## Abstract

Immunotherapy with checkpoint inhibitors (ICIs) has radically changed the landscape of therapeutic opportunities in oncology, but much still needs to be understood from a mechanistic point of view. There is space for further improving tumors’ response to ICIs, as supported by a strong biological rationale. For this achievement a detailed analysis of tumor cell phenotype with functional dissection of the molecular interactions occurring in the TME is required. Galectin-3 is a pleiotropic tumor relevant molecule, which deserves particular attention in immuno-oncology. Due to its ability to finely modulate immune response in vivo, Galectin-3 is a potential target molecule to be considered for overcoming tumor immune escape.

## Background

In the last decade immunotherapy with checkpoint inhibitors (ICIs) has shown unprecedent efficacy in the treatment of patients with different tumors, including aggressive diseases such as melanoma and non-small cell lung carcinoma (NSCLC). This represents a relevant achievement in clinical oncology and expands the hope to prolong the life expectation of patients with metastatic disease. Several pharmaceutical compounds, mostly represented by monoclonal antibodies targeting immunologically relevant inhibitory molecules, such as programmed death-1 (PD-1), programmed death ligand-1 (PD-L1), and cytotoxic T-lymphocyte associated protein-4 (CTLA-4) have been developed and are currently in clinical use. Unfortunately, patients’ response to ICIs is still limited. This fact is not surprising considering the complex scenario of molecular interactions occurring among tumor cells, host cells and components of the tumor microenvironment (TME) that orchestrate tumor immune escape [1, 2]. We focus here on the role played by Galectin-3, a pleiotropic lectin molecule whose immunomodulatory function is relevant. The final aim is to provide the biological rationale for combining ICIs-based immunotherapy with new therapeutic options interfering with mechanisms involved in tumor immune escape.

Tumor infiltrating lymphocytes (TIL) are key actors of tumor immune response. In physiological conditions activation of T-cells is balanced by a fine tuning of inhibitory signals that limit tissue damage and autoimmune reactions, maintaining immune homeostasis. Immune checkpoints (ICs) represent a class of regulatory molecules with inhibitory functions, among which CTLA-4 and PD-1 molecules are the most studied [1]. CTLA-4 is mainly expressed on the surface of Tregs, naive T-cells and effector T-cells and competes with the co-stimulatory receptor CD28 for binding CD80 (B7-1) and CD86 (B7-2) to modulate the strength of the activated T-cell response or to enhance Treg-mediated immunosuppression. On the other side, PD-1 is expressed on the surface of T-cells, B-cells and NK-cells, interacts with PD-1 ligand 1 (B7-H1) and ligand 2 (B7-DC) regulating both central and peripheral tolerance mechanisms. Activation of ICIs pathways combined with the recruitment of suppressive T-cell populations is likely the most common immune escape mechanism adopted by cancer [1, 2].

It is generally accepted that presence of tumor infiltrating lymphocytes (TIL) CD4^+^ and CD8^+^ T-cells in the tumor micro-environment (TME) correlates with better prognosis. These tumors are defined “immunologically hot”. Differently, tumors considered “immunologically cold” show high prevalence of infiltrating Tregs (Foxp3 +) and myeloid derived suppressor cells (MDSCs), which impair CD4^+^ and CD8^+^ T-cell-mediated antitumor response [2]. Immunotherapy with ICIs blocks the inhibitory pathways on effector T-cells and disrupts Treg-mediated immunosuppression [1]. However, specific molecular interactions occurring in the TME provides a fine regulation of tumor immune response, which affects the clinical course of each specific tumor [3, 4].

### The impact of glycobiology on tumor immune escape

The seminal work “The hallmarks of Cancer: the next generation” by Hanahan and Weinberg clearly indicate that analysis of tumor cell genome is not sufficient per se to fully characterize the biological features of aggressive tumors [5]. Very interestingly the proposed hallmarks have been recently revised and integrated with glycobiological data, demonstrating that certain lectin-mediated mechanisms can fine-tune each of the cancer-relevant functions [6].

This information introduces and broadens the knowledge on the mechanisms that underlie tumor growth and progression. As far as tumor immune escape is concerned, experimental works clearly show that specific galectins play a key role in modulating this tumor’s skill [6].

Galectin-3, a β-galactoside-binding protein with a conserved carbohydrate recognition-binding domain, triggers unexpected immunomodulatory functions at cellular and extracellular level. This molecule is an important biological regulator of critical cancer functions, including apoptosis, invasion and metastasis, gene expression, inflammation and fibrosis [3, 4]. Galectin-3 is expressed in several cell types including macrophages, activated T-lymphocytes and epithelial cells, and is highly expressed in different tumor types. This lectin molecule supports and maintains cancer cell survival by activating intracellular and extracellular mechanisms. Galectin-3 contains the NWGR motif, (also worded “death motif”) found in the BH1 domain of Bcl-2 family members and plays as an anti-apoptotic molecule. Several signal transduction pathways and pro-survival processes involving the oncogenes RAS, Bcl-2 and MYC can be activated in Galectin-3 positive tumors [3, 4]. Soluble Galectin-3 released in TME binds specific glycoproteins and glycolipids exposed on the plasma membrane of tumor infiltrating lymphocytes (TIL) modulating their function. Kinase receptors like CD44, CD45, TCR and many other molecules, including cytokines bearing β-galactosyl residues, are potential ligands for Galectin-3. When the binding occurs, the derived lattice modulates the immune response [4, 7, 8].

### Galectin-3 and tumor immune escape

Galectin-3 (MAC-1) is constitutively expressed by macrophages. Tumor infiltrating macrophages (TAMs) represent an important hallmark of tumor progression. Macrophages interact with tumor cells and components of TME acquiring a different phenotype. M1-macrophages have anti-tumor effects, due to intrinsic phagocytosis and enhanced anti-tumor inflammatory reaction. TAMs with M2-phenotype, instead, preferentially secrete galectin-3, angiogenic and anti-inflammatory cytokines, providing an immunosuppressive milieu favoring tumor growth and progression. TAMs may have both M1 and M2 phenotype, depending on cytokine exposure [4]. It is noteworthy that macrophage depletion via colony-stimulating factor-1 receptor (CSF-1R) blockade, improves T-cell infiltration and antitumor activity of PD-1 antagonists in preclinical models of melanoma and breast cancer, suggesting that strategies aimed at inhibiting macrophage response improve the effectiveness of immune checkpoint therapy [9].

**Fig. 1:**
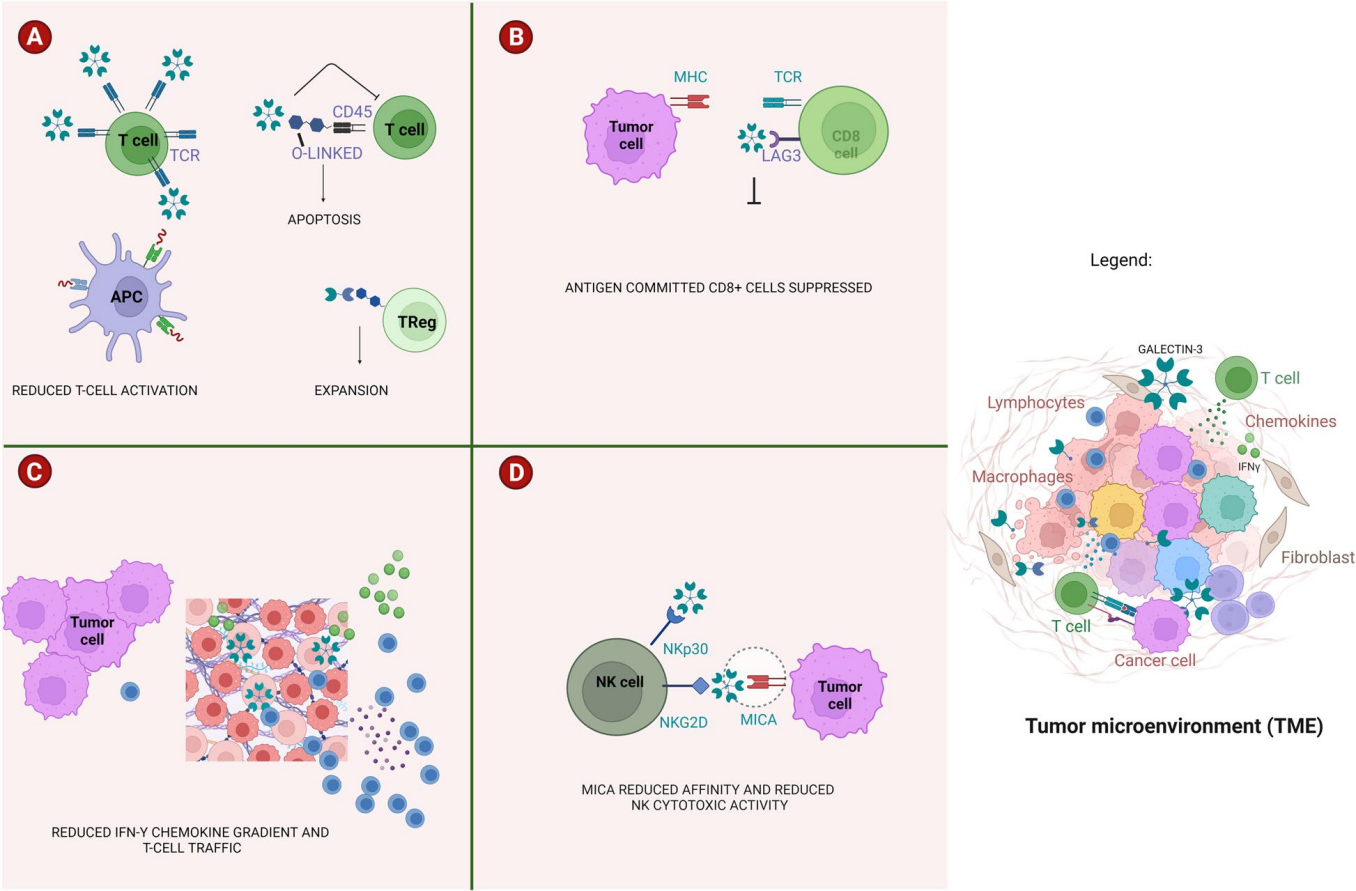
Focusing on Galectin-3—mediated mechanisms of tumor immune escape occurring in TME. Panel **A**) Soluble Galectin-3 interacts with specific glycoligands on TCR. The derived lattice impairs an efficient TCR activation. Apoptosis of T-cells can be triggered when Galectin-3 interacts with specific O-linked sugar residues carried by CD45 receptor. Galectin-3 binding to specific glycoligands expressed on regulatory T-cells promotes expansion of this cell population favoring tumor immune escape. Panel **B** Galectin-3 interacts with the checkpoint molecule LAG3 expressed on antigen committed CD8 + T cells suppressing their function. Panel **C** Galectin-3 released in the TME by macrophages, stromal cells and tumor cells binds to β-galactosyl residues on extracellular matrix components, IFN-γ and glycosylated chemokines. The derived lattice forms a suppressive TME creating a gradient that impairs an efficient exposure of tumor cells to cytotoxic T-cells. Panel **D**) Galectin-3 plays as soluble ligand of NKp30 and NKG2D, two important NK-activating receptors. Galectin-3 reduces the affinity of the major histocompatibility complex class 1-related chain A (MICA) for NKG2D, silencing NK cells

Galectin-3 has a broad range of effects on T-lymphocytes, NK cells, macrophages, neutrophils, and other immune cells. Galectin-3 suppress T-cell function via binding to CD45 N-linked and /or O-linked sugar residues containing β-galactosides. T-cell apoptosis can be triggered when O-linked sugar residues are involved [3, 4]. Interestingly, interaction of Galectin-3 with β-galactosides carried on TCR reduces the activation of this critical receptor, consequently, the cytotoxic activity of CD8 + T-cells results impaired [3, 4] (Fig. 1A).

Furthermore, it has been reported that Galectin-3 binds activated antigen-committed CD8 + T cells via LAG-3 and suppress CD8 + cells cytotoxicity in vitro [3, 4, 10] (Fig. 1B).

The increased number of functional CD8 + T-cells detected in the TME of Galectin-3 KO mice and the expansion of plasmacytoid dendritic cells, which are potent activators of CD8 + cells, compared to Galectin-3 WT-mice, reinforce the evidence of the existing cross-talk between LAG-3 (immune checkpoint) and Galectin-3 [10]. Very recently, treatments of BRAF-mutant melanomas with combination therapies including anti-LAG-3 targeting, showed excellent results [11]. The mechanism by which LAG-3 mediates CD8 + T-cell activity is not fully understood. Interestingly, LAG-3 can be extensively glycosylated and Galectin-3-LAG-3 interaction (via β-galactosides) induces CD8 + T-cell suppression.

The intriguing hypothesis that a Galectin-3 rich -TME could be responsible, at least in part, of tumor immune escape, deserves further investigation [10, 11].

This experimental evidence suggests an alternative mechanism by which Galectin-3 may regulate CD8 + T cell function. Likely, activation of tumor specific CD8 + T cells leads to change the glycosylation repertoire on CD8 + T cell surface, providing additional binding-sites for Galectin-3 mediated immune-regulation.

Experimental evidence also shows that soluble Galectin-3 binds extracellular matrix components and traps glycosylated IFN-γ The resulting lattice prevents the formation of IFN-γ -induced chemokine gradient, which is required for an efficient exposure of cancer cells to activated cytotoxic T-lymphocytes [12] (Fig. 1C).

Moreover, experimental works also demonstrate a direct effect of Galectin-3 in suppressing NK-mediated tumor immune response, by interfering with binding to glycosylated regulatory molecules expressed on cancer cells, which function as NK counter receptors.

Galectin-3 reduces the affinity of MHC-I related chain A (MICA) for NKG2D, an NK-activating receptor, and plays as a soluble inhibitory ligand for NKp30 limiting the cytotoxic activity of NK-cells [13, 14] (Fig. 1D). These effects are mediated by O-glycan sugar residues containing an N-acetylglucosamine branch connected to N-acetylgalactosamine. To further support this finding, genetic down-regulation of Galectin-3 resulted in tumor cells more sensitive to NK cell lysis [14].

All together these experimental data demonstrate that Galectin-3 plays a pivotal role in regulating tumor immune response, maintaining an immune suppressive TME.

The evidence that inhibition of Galectin-3 functions can restore, at least in part, an efficient tumor immune response has a relevant translational value and opens an exciting field of research in both experimental immunology and clinical oncology.

### Translational potential of Galectin-3 in oncology

Considering the regulatory effects that galectin-3 exerts on tumor immune response, several potential clinical applications should be explored.

High Galectin-3 expression in tumor cells is associated with poor prognosis and tumor progression in non-small cell lung cancer (NSCLC). Moreover the analysis of galectin-3 expression in metastatic NSCLCs candidate for immunotherapy with ICIs, could have a high predictive value of therapeutic response [15]. Validation of the “galectin-3 signature" as predictive marker of tumor responsiveness to ICIs may be clinically relevant and promises a better selection of patients candidate to immunotherapy. To this aim an immunoPET approach, which use humanized galectin-3 probes for tumor imaging in vivo*,* is going to be translated in the clinical setting. This imaging approach will avoid invasive diagnostic procedures, unnecessary therapies and relevant social costs [16, 17].

## Conclusion

TCR engagement, co-receptor activation and cytokine production are historically considered the key events of immune response. In the last decade research in glycobiology contributed to change the paradigm. Specific molecules with lectin function play as fine regulators of the immune response at least in tumors. The preliminary finding that expression of galectin-3 in metastatic NSCLCs shows a predictive value of tumor responsiveness to ICIs is clinically important and deserves validation in multicenter studies [15]. At least theoretically, this specific signature may be biologically relevant for different types of tumors candidate to ICIs-based immunotherapy. The availability of immunoPET technology, which use galectin-3 humanized probes for tumor imaging in vivo*,* promises a prompt selection of patients with metastatic tumors candidate to immunotherapy. In case of Galectin-3 positive tumors, a combined immunotherapy with galectin-3 inhibitors could be finally considered, for improving the efficacy of immunotherapy and overcome tumor immune escape.

## Data Availability

The dataset supporting the conclusions of this article is included within the article.

## Abbreviation

ICIs: Immunotherapy with checkpoint inhibitors
TME: Tumor microenvironment
PD-1: Programmed death-1
PDL-1: Programmed death ligand-1
CTLA-4: Cytotoxic T-lymphocyte associated protein-4
NSCLC: Non-small cell lung carcinoma
TIL: Tumor infiltrating lymphocytes
TAMs: Tumor-associated macrophages
MDSCs: Myeloid derived suppressor cells
MICA: MHC-I related chain A
ImmunoPET: Positron emission tomography
